# Evaluation and Exploration of Favorable QTL Alleles for Salt Stress Related Traits in Cotton Cultivars (*G*. *hirsutum* L.)

**DOI:** 10.1371/journal.pone.0151076

**Published:** 2016-03-04

**Authors:** Lei Du, Caiping Cai, Shuang Wu, Fang Zhang, Sen Hou, Wangzhen Guo

**Affiliations:** State Key Laboratory of Crop Genetics & Germplasm Enhancement, Hybrid Cotton R & D Engineering Research Center, Ministry of Education, Nanjing Agricultural University, Nanjing 210095, China; USDA-ARS-SRRC, UNITED STATES

## Abstract

Soil salinization is one of the major problems in global agricultural production. Cotton is a pioneer crop with regard to salt stress tolerance, and can be used for saline-alkali land improvement. The large-scale detection of salt tolerance traits in cotton accessions, and the identification of elite quantitative trait loci (QTLs)/genes for salt-tolerance have been very important in salt tolerance breeding. Here, 43 advanced salt-tolerant and 31 highly salt-sensitive cultivars were detected by analyzing ten salt tolerance related traits in 304 upland cotton cultivars. Among them, 11 advanced salt-tolerance and eight highly salt-sensitive cultivars were consistent with previously reported results. Association analysis of ten salt-tolerance related traits and 145 SSRs was performed, and a total of 95 significant associations were detected; 17, 41, and 37 of which were associated with germinative index, seedling stage physiological index, and four seedling stage biochemical indexes, respectively. Of these associations, 20 SSR loci were simultaneously associated with two or more traits. Furthermore, we detected 117 elite alleles associated with salt-tolerance traits, 4 of which were reported previously. Among these loci, 44 (37.60%) were rare alleles with a frequency of less than 5%, 6 only existed in advanced salt-tolerant cultivars, and 2 only in highly salt-sensitive cultivars. As a result, 13 advanced salt-tolerant cultivars were selected to assemble the optimal cross combinations by computer simulation for the development of salt-tolerant accessions. This study lays solid foundations for further improvements in cotton salt-tolerance by referencing elite germplasms, alleles associated with salt-tolerance traits, and optimal crosses.

## Introduction

With frequent extreme weather conditions and climate change, there is the urgent need to breed new crop varieties that are adaptable to adverse environments such as drought, heat, submergence, and high salinity. Soil salinization is one of the major problems challenging global agricultural production. It is reported that more than 6% of the 800 million hectares of land throughout the world are salt affected [1]. To effectively solve this, identifying keys genes for abiotic stress tolerance at a genome-wide level and mining elite crop resources are critical procedures for abiotic stress-resistant breeding.

Cotton (*Gossypium* spp.) is not only an important economic crop worldwide, but is also a pioneer crop in saline-alkali land. Cotton can be used for saline-alkali land improvement, which providing improved farmland for future food production. Nevertheless, no cotton cultivar is suitable for planting in large areas of saline-alkaline land with high concentrations of salt. There has been little research into the identification of large-scale salt tolerance traits in cotton accessions, or quantitative trait loci (QTLs) mapping for salt tolerance. Jia et al. [2] reported three markers associated with salt tolerance through association mapping (AM) methods using 323 *Gossypium hirsutum* germplasms and 106 microsatellite markers. Saeed et al. [3] (2014) analyzed the marker association for salt tolerance in cotton (*Gossypium hirsutum* L.) germplasms from the US and diverse regions of China. However, the mining and identification of QTLs/genes related to salt tolerance in cotton needs to be strengthened.

There are several methods that can be used for the evaluation of germplasms and elite QTLs/genes. Factor analysis is commonly used to evaluate the status of each material in a group, through analyzing a large number of samples and major relevance indicators [4–7]. Association analysis is based on linkage disequilibrium (LD) and uses a sample of lines from broader breeding populations, unrelated by any specific crossing design [8]. Molecular breeding by design, first presented by Peleman and van der Voort [9], can be achieved by following three steps: (1) Mapping loci involved in all agronomically relevant traits; (2) Assessment of the allelic variation at those loci; (3) Breeding by design. Molecular breeding by design greatly increases predictability in conventional breeding, leading to the progression from “phenotypic breeding by experience” to “genotypic breeding by prediction” with high breeding efficiency and effectiveness [10].

Here, using these three analysis methods, we performed large-scale identification of salt tolerance traits in cotton cultivars and used association analysis of markers and traits related to salt tolerance at a whole genome level to explore elite germplasms and QTLs/genes for salt tolerance. In this study, the phenotypic data of ten different traits related to salt tolerance were used to comprehensively evaluate the salt tolerance of 304 upland cotton cultivars by factor analysis. Elite cultivars tolerant to salt stress were thus identified, and further association analysis of salt tolerance traits and 145 polymorphic microsatellite markers was performed. By integrating the comprehensive evaluation of salt tolerance traits with the numbers of plants carrying elite alleles for each cultivar, a few advanced salt-tolerant cultivars were selected to assemble the optimal cross patterns by computer simulation for the development of salt-stress resistant accessions. This study provides molecular information and elite germplasms for salt-tolerance improvement breeding via marker-assisted selection (MAS), and also provides optimal combinations for further salt tolerance breeding in cotton.

## Materials and Methods

### Plant materials

A total of 304 upland cotton cultivars (S1 Table) were selected for this experiment. These cultivars were made available from the cotton germplasm collection at the Hybrid Cotton R&D Engineering Research Center, Ministry of Education (Nanjing, China) and the Institute of Cotton Research, Chinese Academy of Agricultural Science (CRI-CAAS). Of these upland cotton cultivars, 294 were collected from China and 10 were introduced from America. Chinese cultivars could be divided into four different ecological areas according to the ecological characteristics of the cotton growing regions: the Yellow River (177), the Yangtze River (78), the Northwestern inland region (23), and the Northern China region, specifically, the early maturation area (16).

In 2012, the 304 upland cotton cultivars were planted in the Jiangpu experimental station of Nanjing Agricultural University, Nanjing, Jiangsu Province, China, for reproduction and sampling, and grown using normal field practices. All necessary permits for the field evaluations of these accessions were obtained from Nanjing Agricultural University, China. All the field evaluations were not relevant to human subject or animal research. Therefore, they did not involve any endangered or protected species.

Genomic DNA was isolated from all 304 cotton cultivars using the methods described by Paterson et al [11]. Simple-sequence repeat polymerase chain reaction (SSR-PCR) amplifications were performed using an Applied Biosystems Veriti Thermal Cycler (ABI, USA), and electrophoresis of the products was performed according to the method described by Zhang et al [12].

Based on the high-density genetic linkage map constructed in our laboratory [13], SSRs at ca. 10 cM intervals in each chromosome were selected to ensure an even covering of the tetraploid cotton genome. Furthermore, SSR markers related to salt-tolerance QTLs reported in previous studies [14–15] were also selected for screening for polymorphisms in the 304 upland cotton cultivars. Of these 381 SSRs, 145 showed polymorphisms in 26 chromosomes of the cotton genome. All SSR information can be downloaded from http://www.CottonGen.com/. Amplification products were scored as either a present (1) or an absent (0) band. The alleles were coded A, B, C, and so on according to their molecular weight.

### Investigation of salt tolerance traits in upland cotton cultivars

An experimental design modified from the methods of Zhang et al [16] was used for salt tolerance evaluation at cotton germination and seedling stages. In 2013, a germination experiment was carried out with three replications, and 100 cotton seeds were used for each treatment. The same sized seeds for each cultivar were surface sterilized, transferred into 9 cm sterile Petri dishes on filter paper, and were then wetted with 4 mL distilled water (control) or 150 mM NaCl. To prevent infection and evaporation, all of the plates were closed with parafilm, and the filter paper and solution were replaced every 2 days. The Petri dishes were incubated in a growth chamber at 28°C, 80% relative humidity and with no light. The germination rate and germination percentage, which represented the germination capacity 3 days and 7 days after treatment, were calculated.

Seeds from 304 cotton cultivars were surface sterilized and allowed to grow in 1/2 diluted Hoagland solution [17]. At the developmental period where two leaves had formed, four seedlings per cultivar, with three repeats, were grown in boxes (37 × 55 × 13 cm) for NaCl treatment, and the same were grown with only 1/2 diluted Hoagland solution as a control. The experimental plants were exposed to salinity by adding NaCl to the growth medium in 50 mM increments every 24 h, until 150 mM was reached. After 15 days, at the stage of five to six leaves, 3 plants were selected and their plant height, shoot dry matter weight, and root dry matter weight were measured. Chlorophyll content was measured using a SPAD-502 [18], with three plants per treatment, and three measurements per leaf (top second leaf) were averaged per plant. The superoxide dismutase, peroxidase, and catalase enzyme activities were estimated using the methods previously described by Beauchamp and Fridovich [19–21]. Lipid peroxidation was estimated via the plants’ malondialdehyde content [22].

### Data analysis

#### Genetic diversity

The relative phenotypic data of ten salt-tolerance traits were calculated using the following formula: (phenotypic effects value under salt stress / phenotypic effects value under well-watered control) × 100%. Standard deviations, coefficients of variation and correlation analyses were estimated using SPSS18.0 (http://www.spss.com.cn/). POWERMARKER v 3.25 software (http://statgen.ncsu.edu/powermarker/) [23] was used to estimate the number of alleles per marker, the gene diversity, and the polymorphism information content (PIC) of 145 SSRs for the tested cotton accessions.

#### Factor analysis of salt-tolerance traits

Factor analysis is a scientific and objective method which is often used for factor extraction and comprehensive evaluation. This method studies the spatial distribution of the research objects so as to identify groupings and the relationships between them. In order to determine whether the selected variables for factor analysis were related, the Kaiser-Meyer-Olkin (KMO) measurement of sample adequacy and the Bartlett Test of Sphericity (BTS) were calculated using SPSS software (http://www.spss.com.cn/). Factors were chosen based on the cumulative-contribution-rate-more-than-85% rule [4–7]. Finally, the factor scores were used for the comprehensive evaluation of the salt-tolerance of each cultivar.

#### Association mapping

Population structure analysis: The model-based STRUCTURE v 2.3.3 software (http://pritch.bsd.uchicago.edu/software. html) [24] was used to infer the population structure of 304 cotton cultivars (K = 2 to K = 10, with three runs at each K) using a burn-in of 10,000 and a run length of 100,000. The most likely number of clusters (K) was selected by comparing the LnP (D) and ΔK [25]. Q-matrix was derived for the subsequent AM.

Linkage disequilibrium: LD was evaluated for each pair of SSR loci using the TASSEL 2.0.1 software package (www.maizegenetics.net/bioinformatics/tasselindex.htm) [26]. All pairs of adjacent loci within the same LD chromosome were referred to as an LD block [27]. LD was detected using a modified D’ method [28]. Statistical significance (P-values) of D’ for each SSR pair was determined with 100,000 permutations.

Association mapping: Mixed linear models (MLMs) and general linear models (GLMs) were used to construct salt-tolerance related traits association tests using the TASSEL 2.0.1 software package (www.maizegenetics.net/bioinformatics/tasselindex.htm) [26]. The MLM association test was performed by the simultaneous accounting of multiple levels of population structure (Q-matrix) and relative kinship among the individuals (K-matrix) according to the methods described by Yu et al [29]. The GLM association test only estimated the Q-matrix. A data file of the Q-matrix was created using Structure 2.3.3, and the K-matrix was created by calculating kinship values using TASSEL.

#### Exploration and utilization of favorable alleles

Exploration of favorable alleles: An allele’s effect (ai) on salt-tolerance traits was evaluated by comparing the traits of the specific allele and the average value in 304 cotton cultivars, following methods reported previously [30–31].

Simulation in molecular breeding by design: Simulation experiments can provide a reference for the combination and assortative mating of favorable alleles. Using favorable receptor parents with a large number of favorable alleles, simulation of the breeding process was carried out using single cross, three-way cross and four-way cross methods with other cultivars. In this study, 13 upland cotton cultivars with a large number of favorable alleles were selected as receptors for the simulation of molecular design breeding to determine the conditions that pyramid the largest number of favorable alleles.

## Results

### Identification of salt tolerance in 304 upland cotton cultivars

#### Phenotypic diversity analysis

In 304 upland cotton cultivars (S1 Table) treated with 150 mM NaCl and in well-watered controls, 10 traits related to salt tolerance were measured: germination rate (GR) and germination percentage (GP) at germinating stage, plant height (PH), shoot dry matter (SDM), root dry matter (RDM), chlorophyll content (CC), malondialdehyde (MDA) content, and superoxide dismutase (SOD), peroxidase (POD) and catalase (CAT) enzyme activity at seedling stages. The mean values, ranges, standard deviations, and coefficients of variation for the ten salt-tolerance related traits are shown in Table 1.

**Table 1:** Statistics of various traits related to salt tolerance with 150 mM NaCl (Salt stress treatment) or 0 mM NaCl (Control) in two-leaf seedling in cotton cultivars.

Trait	Salt stress treatment*			Control		
	Mean(range)	SD	CV%	Mean(range)	SD	CV%
Chlorophyll content, CC, SPAD	27.23(17.30–34.90)	3.19	11.71	31.64(24.00–39.05)	2.60	8.21
Plant height, PH, cm	11.5(2.07–21.20)	4.00	34.75	21.11(8.60–32.55)	3.98	18.85
Root dry matter, RDM, g	0.06(0.02–0.17)	0.03	47.12	0.08(0.02–0.40)	0.05	59.55
Shoot dry matter, SDM, g	0.37(0.07–1.03)	0.15	40.13	0.65(0.14–1.67)	0.26	39.68
Germination rate, GR, %	60.32(11.67–100.00)	20.89	34.63	87.35(30.00–100.00)	12.20	13.97
Germination percentage, GP, %	79.98(33.33–100.00)	13.14	16.43	91.2(43.33–100.00)	10.01	10.98
SOD activity, U/g FW	384.37(34.80–700.67)	143.96	37.45	601.58(131.18–918.44)	162.43	27.00
POD activity, U/(g•min)	17579.61(5324.51–33617.38)	6476.77	36.84	21553.98(8146.63–32303.18)	4981.56	23.11
CAT activity, U/(g•min)	39.99(2.91–138.18)	22.16	55.42	82.48(6.35–231.45)	39.32	47.68
MDA content, nmoL/g FW	20.92(2.59–156.66)	17.64	84.32	9.88(1.68–23.25)	4.34	43.96

*SD: standard deviations; CV: coefficient of variation

The mean and extreme values of nine salt-tolerance traits were lower in cultivars under salt stress treatment than in controls, however, MDA content was higher. This is consistent with previous findings [14, 32–41]. The coefficients of variation (CV%) of the ten salt-tolerance traits ranged from 11.71 (chlorophyll content) to 84.32 (MDA content) under salt stress, and from 8.21 (chlorophyll content) to 84.32 (root dry matter) in controls. We also compared CV% for all tested traits under salt stress treatment and control conditions, and found that for all salt-tolerance related traits except root dry matter, CV% under salt stress was larger than that under control conditions; implying that the cotton accessions used in this study represented a large range tolerance variation under salt stress treatment.

Further, the relative values of the ten salt-tolerance traits were calculated using the ratio of the phenotypic effects value under salt stress conditions and the phenotypic effects value under well-watered control conditions, and correlation analysis was performed to elucidate the relationships between the ten salt-tolerance traits (S2 Table). As a result, a significant positive correlation was found between the relative germination rate and the relative germination percentage. There were also significant positive correlations in relative CC, relative PH, relative RDM and relative SDM.

#### Comprehensive evaluation of salt-tolerance

In order to study the relationship between ten salt-tolerance related traits and the salt tolerance of 304 cotton cultivars, factor and cluster analyses were performed. First, the Kaiser-Mayer-Olkin (KMO) index was calculated and Bartlett's test was performed to analyze whether the original data set was suitable for factor analysis. The KMO value was 0.627 (>0.5), and the Bartlett's Test value was (χ^2^ = 1052.464 > χ^2^_0.01, 45_ = 69.957, and Sig. 0.000 < 0.05) [4], which showed that the raw data were suitable for factor analysis.

Based on the cumulative-contribution-rate-more-than-85% rule, a six-factor solution that accounted for 86.31% of the total variance was obtained. Factor 1 represented the seedling stage physiological index factor, which included the relative chlorophyll content (RCC), relative plant height (RPH), relative root dry matter (RRDM) and relative shoot dry matter (RSDM). Factor 2 was termed the germinative index factor, and included the relative germination rate (RGR) and relative germination percentage (RGP). Factors 3–6 represented the seedling stage biochemical index factors, and included the relative SOD (RSOD) activity, relative POD (RPOD) activity, relative CAT (RCAT) activity, and relative MDA (RMDA) content, respectively. The results of the factor analyses were consistent with the correlation analysis, where a significant positive correlation was detected between the two germinative indexes, and the four seedling stage physiological indexes.

Further, the F factor composite score for the salt-tolerance of each cotton cultivar was obtained, based on the six F factors (S1 Table). By cluster analysis, the 304 upland cotton cultivars were divided into four groups based on their salt tolerance. 43 cultivars were advanced salt-tolerant plants with an F factor ranging from 0.496 to 1.100, 114 cultivars had medium salt-tolerance (F factors ranging from 0.000 to 0.458), 116 cultivars were salt-sensitive (F factors ranging from -0.565 to -0.01), and 31 cultivars were highly salt-sensitive (F factor ranging from -1.15 to -0.582).

### Association analysis for SSR loci and salt-tolerance traits in upland cotton

#### Genetic diversity analysis

Based on our previously published genetic linkage map information from *G*. *hirsutum* × *G*. *barbadense* [13], and simple sequence repeats (SSR) markers related to salt-tolerance QTLs reported in previous studies [14, 15], a total of 381 SSRs with broad genome-wide coverage were used to screen the polymorphisms of 12 randomly selected upland cotton cultivars from different ecological regions. Of these markers, 145 SSRs showed polymorphisms in 26 chromosomes; 68 and 77 SSRs in the A- and D-subgenomes, respectively. The number of SSR markers per chromosome ranged from 3 to 8 (S3 Table).

The 145 SSRs were used to further screen the polymorphisms in 304 upland cotton cultivars. A total of 640 alleles were obtained, with an average of 4 alleles per locus (ranging from 2 to 12 alleles per locus). The average genetic diversity was 0.365 (ranging from 0.007 to 0.781). The average polymorphism information content (PIC) was 0.319 (ranging from 0.007 to 0.747) (S3 Table).

#### Population structure analysis

**Fig 1:**
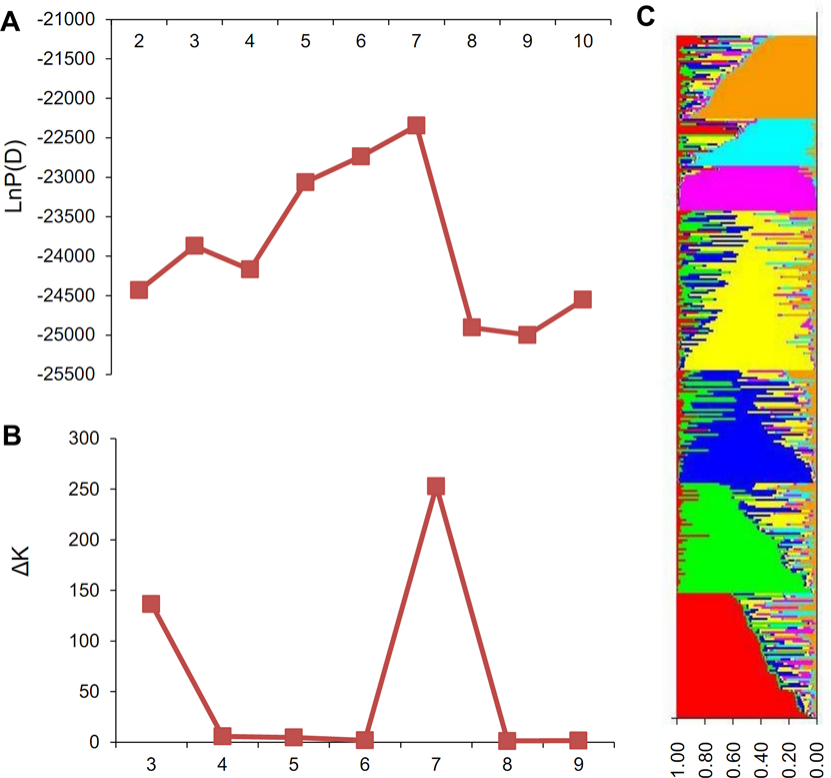
Population structure analysis in 304 cotton cultivars/accessions. (A) The most likely number of clusters (K) by LnP(D) analysis; (B) The most likely number of clusters (K) by ΔK analysis; (C) Population structure in 304 cotton cultivars/accessions (K = 7).

The population structure of the 304 upland cotton cultivars was analyzed by STRUCTURE V2.3.3 software based on 145 SSR markers. The results showed that the most likely number of subpopulations (K) was seven, according to maximum LnP (D) values and the maximum ΔK value (Fig 1A and 1B), which indicated that the entire population could be divided into seven subpopulations (Fig 1C). The corresponding Q matrix at k = 7 was then used for the subsequent association mapping.

Correlation analyses were used to study the associations between the seven sub-population clusters and five geographic eco-types (the Yellow River Valley, the Yangtze River Valley, Northern China area, Northwestern inland and accessions from the US) in the 304 upland cotton cultivars. We found that the population structure was associated with the geographic eco-type (χ^2^ = 81.98 > χ^2^_0.01, 24_ = 51.18), indicating that the classification of geographic eco-types in the natural population was of a sound genetic basis.

#### Linkage disequilibrium

Linkage disequilibrium (LD) analysis of 145 SSRs was estimated using the TASSEL 2.0.1 software package. The LD distribution of the 145 SSRs in the 304 upland cotton cultivars is shown in S1 Fig. Among the 10,440 SSR locus combinations (145*144/2), 2,328 (22.3%) showed significant LD (P<0.05) in the 304 upland cotton cultivars. The interchromosomal LD was 2,218, which was higher than the intrachromosomal LD of 110.

#### Association analysis

At the p<0.05 level, a total of 95 significant associations were detected between 60 SSRs and ten salt-tolerance related traits using mixed linear model (MLM) and general linear model (GLM) analyses (Table 2). These SSRs were distributed in almost all cotton chromosomes except Chro.D4. Among these, most of the associations (87 of 95) were detected in both the GLM and MLM, 2 associations were detected only in the GLM, and 6 were detected only in the MLM. From the MLM test, the average explained phenotypic variation was 4.49%, and the overall values ranged from 2.07% to 13.7%. Of these associations, 17 were associated with the germinative index (involving 10 SSRs), 41 were associated with the seedling stage physiological index (involving 26 SSRs), and 37 were associated with the four seedling stage biochemical indexes (involving 33 SSRs). The greatest and the lowest numbers of marker-trait associations were 17 for RMDA and 1 for RCAT, respectively. The number of associations for the other eight salt-tolerance traits ranged from 8 to 12.

**Table 2:** Marker loci associated with traits and their explained phenotypic variation in cotton cultivars.

Locus	Position(cM)	Traits related to salt tolerance*									
		RCC	RPH	RRDM	RSDM	RSOD	RPOD	RCAT	RMDA	RGR	RGP
NAU2437	A1(13.28)			6.81					**8.72**	8.45	9.14
NAU7049	A1(33.484)					**3.88**					
NAU2419	A1(89.554)										4.84
BNL3424	A2(107.067)								2.75		
NAU2161	A3(6.031)	**7.06**									
NAU483	A3(28.185)									3.81	
NAU7182	A4(20.606)		2.67	**5.04**	3.64						
NAU3592	A4(115.829)	**6.3**	**8.52**						4.18		
NAU934	A5(19.745)								4.26		
NAU2561	A5(55.633)								2.62		
BNL3452	A5(183.628)								**13.7**		
NAU2679	A6(58.824)								**11.85**		
NAU2995	A7(52.324)					2.51					
BNL3792	A8(47.75)		2.8M								
Gh486	A9(10.75)								3.74M		
BNL1414	A9(95.796)		3.31	**5.74**	**5.38**						
NAU462	A9(112.13)								**5.04**		
NAU2508	A10(0.51)			3.75			3.49				
NAU4921	A10(12.65)			2.42G			2.56				
NAU5166	A10(98.924)								**3.76**		
NAU5192	A11(3.272)								**6.64**		
BNL1231	A11(162.784)					2.18					
NAU1151	A12(98.908)								**5.78**		
NAU3398	A13(52.406)									4.92	3.75M
NAU2300	A13(97.812)					2.07	2.12	2.48			
JESPR204	A13(105.781)				5.53		**5.56**				
JESPR152	D1(17.336)		**3.9**		3.2M						**6.63**
NAU2901	D1(23.449)								3.17		
TME03	D1(77.181)						2.86				
NAU458	D1(124.81)					**4.03**					
CIR246	D2(0)			4.42							
NAU2173	D2(63.802)	5.38									
NAU5027	D2(72.797)								**4.79**		
NAU2190	D2(93.309)		3.59								
NAU7024	D3(58.626)									4.93	6.13
NAU3639	D3(68.254)			2.44							
NAU1042	D5(121.466)			3.73							
BNL3594	D6(10.127)								**8.04**		
NAU7209	D6(101.047)					4.11					
BNL3436	D6(114.173)					**4.65**	3.33		3.77		
NAU2714	D6(121.89)		**3.69**	2.48							
BNL3359	D6(142.146)	2.43	2.59		2.5						
NAU3911	D7(31.898)			2.57	2.38					2.63	2.45
BNL1694	D7(55.78)		4.63							5.24	**7.42**
NAU6752	D7(75.348)					**4.66**					
NAU3053	D7(99.873)	3.27									
NAU493	D7(115.416)	2.59									
NAU1350	D8(59.972)									2.33	2.74
NAU1369	D8(108.998)		3.63G								
NAU3986	D9(57.431)								4.26		
JESPR208	D9(116.611)		4.8	**5.61**	**6.31**						
NAU5189	D9(137.181)		2.77M	**6.04**	3.35M						
NAU6755	D10(92.245)	2.68									
BNL1404	D11(43.704)						**4.79**				
Gh508	D11(55.82)						2.8				
NAU5418	D11(77.269)	5.91									
NAU429	D11(162.789)						**7.13**				
NAU3862	D12(30.497)						2.69				
BNL3537	D12(93.32)						3.71			**5.35**	**5.01**
NAU2697	D13(34.095)	**6.04**									

*Abbrevation for 10 traits related to salt tolerance is relative chlorophyll content (RCC), relative plant height (RPH), relative root dry matter (RRDM), relative shoot dry matter (RSDM), relative SOD activity (RSOD), relative POD activity (RPOD), relative CAT activity (RCAT) and relative MDA content (RMDA), relative germination rate (RGR), relative germination percentage (RGP), respectively. P<0.05 is indicated in general case; P<0.01 is indicated in boldface; G means significance detected only in general linear model (GLM) test; M means significance detected only in mixed linear model (MLM) test; No marked values means detected both by MLM and GLM tests.

We also found that 20 SSR loci were simultaneously associated with two or more traits. Among them, 2, 11, and 7 loci were simultaneously associated with 4, 3, and 2 salt-tolerance related traits, respectively. Most of the traits associated simultaneously with the common markers belonged to the same factor type identified by the factor analysis. For example, seven SSRs (NAU1350, NAU3911, BNL3537, NAU7024, BNL1694, NAU2437, and NAU3398) were simultaneously associated with RGR and RGP in the germinative index factor. Five SSRs (NAU2714, NAU7182, JESPR208, BNL1414, and NAU5189) were simultaneously associated with RPH and RRDM, and five SSRs (NAU7182, BNL1414, JESPR208, JESPR152, and NAU5189) were simultaneously associated with RPH and RSDM in the seedling stage physiological index factor. In the four seedling stage biochemical index factors, two SSRs were simultaneously associated with three traits: BNL3436 with RSOD, RPOD, and RMDA, and NAU2300 with RSOD, RPOD and RCAT.

### Mining of favorable alleles and breeding utilization

#### Mining of favorable salt-tolerance alleles

A total of 95 marker-trait associations were employed to explore elite alleles. As a result, 117 elite alleles associated with salt-tolerance related traits were detected, with all allele effects (ai) and typical materials listed in S4 Table. The highest and lowest numbers of elite alleles were 35 for RMDA and one for RCAT, respectively. The number of elite alleles for the other eight salt-tolerance traits ranged from 11 to 19. We also found that some elite alleles were simultaneously associated with more than two traits. Among these favorable alleles, the most positive phenotypic effects for the ten salt-tolerance traits ranged from 0.0918 (RCAT) to 2.1461(RMDA). Of these alleles, 44 (37.60%) were rare alleles with a frequency of less than 5% (they existed in ≦15 cultivars in all 304 cotton cultivars).

We also compared the alleles in 43 advanced salt-tolerance cultivars and 31 highly salt-sensitive cultivars. The results showed that 6 positive alleles and 4 negative alleles (NAU1369-G, CIR246-C, BNL3424-B, and NAU5027-B) existed only in advanced salt-tolerant cultivars, 2 positive alleles and 9 negative alleles (BNL1231-C, BNL3452-C, JESPR152-B, NAU462-G, NAU1042-G, CIR246-G, NAU2173-G, NAU2419-C, and NAU2697-G) existed only in highly salt-sensitive cultivars. Of the six elite alleles that existed only in advanced salt-tolerant cultivars, NAU5189-G was associated with RPH, RRDM, and RSDM; NAU2161-H and NAU2173-C were associated with RCC; and NAU483-B, NAU2190-C, and NAU7049-B were associated with RGR, RPH, and RSOD, respectively. The two elite alleles that existed only in highly salt-sensitive cultivars were NAU2419-G and NAU5192-D, which were associated with RGP and RMDA, respectively. Of the eight positive elite alleles, five were rare alleles. The three that were not rare were NAU483-B, NAU2161-H, and NAU5189-G.

We further mined the 304 accessions carrying the elite alleles (S1 Table). The average number of elite alleles was 35.99, and the overall numbers ranged from 23 (M-8124-1159) to 46 (Yan1113). There were 176 accessions (57.89%) contained elite alleles more than 35.99 in average, implying a better salt stress tolerance in most cotton cultivars.

#### Simulation of molecular breeding by design

In order to develop the stronger salt tolerance accessions suitable for different ecological areas, crossing was simulated for molecular breeding by design. In theory, we assumed that the more stacking the favorable alleles, the better the salt tolerance of accessions. By integrating the comprehensive evaluation of salt-tolerance with the number of elite alleles carried in each cultivar, we selected 13 cultivars as receptor parents to conduct molecular breeding by design. Of them, 11 cultivars, Zhongmiansuo44, Xuzhou58, Dongting1, Zhongmiansuo3, Emian14, Ejing92, Zhongmiansuo43, Yumian17, Sumian20, Xin80477, and Lu458, were advanced salt-tolerance cultivars with more than 40 elite alleles, and two cultivars, Yan1113 and Sumian8, were medium salt-tolerance cultivars with 46 and 45 elite alleles, respectively. Further, these 13 cotton cultivars were also derived from three different ecological areas: 6 cultivars, Yan1113, Sumian8, Dongting1, Emian14, Ejing92, and Sumian20, were from the Yangtze River cotton planting region, cultivar Xin80477 was from the Northwestern inland cotton planting region, and the remaining six cultivars were planted widely in the Yellow River region.

The above-mentioned 13 receptor parents were used to simulate the breeding process by single crossing, three- way crossing and four-way crossing with other cultivars. This produced 303 single cross combinations, 45,753 three-way cross combinations and 4 590,551 four-way cross combinations for each receptor parent. The sum of pyramiding favorable alleles was computed for each combination (Table 3). For the 13 receptor parents, the average number of elite alleles was 41.31, ranging from 40 to 46. Through the single, three-way, and four-way crosses, the mean of the pyramiding elite alleles rose to 51.46, 55.62, and 57.85, respectively. The number of potential cross combinations over the mean value was 41 for single crosses (>51.46), 102 for three way crosses (>55.62), and 319 for four way crosses (>57.85). The optimal cross and the number of elite alleles for the 13 cultivars are listed in S5 Table. By combining the ecological characterizations of both receptors and donors with the simulation results, the ideal parents and cross combination can be selected for pyramiding the largest number of elite alleles. However, the resistance ability to salt stress for these germplasms remains to be confirmed in field experiments.

**Table 3:** Molecular breeding by design for pyramiding the favorable alleles by assembling the combinations with 13 salt-tolerant cultivars as receptor and other 303 accessions as donors.

Receptor	Geographic eco-types region	No. of elite alleles	Single cross Mean(range)	Three-way cross Mean(range)	Four-way cross Mean(range)
Yan1113	the Yangtze River	46	49.85(47–54)	51.46(47–57)	52.54(47–59)
Sumian8	the Yangtze River	45	47.59(45–54)	49.46(45–57)	50.84(45–59)
Dongting1	the Yangtze River	41	45.51(41–53)	48.07(42–56)	49.80(42–58)
Sumian20	the Yangtze River	40	45.44(41–51)	48.04(42–56)	49.73(42–58)
Ejing92	the Yangtze River	40	44.68(41–51)	47.54(41–55)	49.43(41–57)
Emian14	the Yangtze River	40	43.77(40–49)	46.47(40–55)	48.45(41–58)
Zhongmiansuo44	the Yellow River	43	46.73(44–53)	48.76(44–56)	50.13(44–58)
Xuzhou58	the Yellow River	42	45.67(43–51)	48.06(43–55)	49.71(43–57)
Lu458	the Yellow River	40	45.37(41–51)	48.15(41–55)	49.87(42–58)
Yumian17	the Yellow River	40	44.70(40–50)	47.34(41–55)	49.13(42–58)
Zhongmiansuo3	the Yellow River	40	44.58(41–50)	47.17(41–55)	48.97(42–57)
Zhongmiansuo43	the Yellow River	40	44.49(40–51)	47.18(41–55)	48.99(41–57)
Xin80477	the Northwestern inland	40	46.37(42–51)	49.32(42–56)	50.99(42–58)

## Discussion

Soil salinization is a serious global problem affecting agricultural development and ecological environments. Cotton is a salt-tolerant crop, and upland cotton comprises 95% of cultivated cotton worldwide. Salt tolerance varies greatly among different upland cotton varieties. Previous studies on cotton salt-tolerance have focused on a given period of development, for example the germination or seedling stage, and only a small number of indexes have been identified in cotton varieties [16, 42–45]. The large-scale identification of salt tolerance traits in cotton accessions, and the exploration of elite salt-tolerant QTLs/genes and germplasms, need to be studied systematically to improve cotton abiotic stress-resistance.

### Factor and cluster analysis is an effective method for mining main factors and accessions related to stress tolerance

In this study, we investigated the salt tolerance of 304 upland cotton varieties, comprising 294 upland cotton cultivars collected from four different ecological areas in China and 10 introduced from America. A total of 10 traits related to salt-tolerance were measured; two traits detected at the germinating stage (germination rate and germination percentage), and 8 traits detected at the seedling stages (plant height, shoot dry matter weight, root dry matter weight, chlorophyll content, MDA content, SOD, POD and CAT enzymes activity). Based on factor and cluster analyses [4–7], a comprehensive evaluation of the salt-tolerance of each cultivar was completed by integrating the traits associated with the germination and seedling stages.

Factor analysis is a development of the principal component analysis (PCA) method. It reduces the number of primary variables by calculating a smaller number of new variables, called factors [46–47]. Through factor analysis, the ten indexes were combined into six factors belonging to three index factors: the physiological index factor in the seedling stage (Factor 1: RCC, RPH, RRDM and RSDM); the germinative index factor (Factor 2: RGR and RGP); and the biochemical index factor in the seedling stage (Factors 3–6: RSOD, RPOD, RCAT, and RMDA, respectively). Factor analysis was highly consistent with correlation analysis, and there was a significant positive correlation between the two germinative indexes, and among the four seedling stage physiological indexes.

Through cluster analysis, 304 upland cotton cultivars were divided into four groups: 43 advanced salt-tolerance cultivars, 114 medium salt-tolerance cultivars, 116 salt-sensitive cultivars, and 31 highly salt-sensitive cultivars. Among the 43 advanced salt-tolerance cultivars and 31 highly salt-sensitive cultivars, 11 and 8 had been reported in the previous studies, respectively [15, 33, 35, 36, 38–41, 48].

In summary, factor and cluster analyses are useful methods for validating the salt tolerance of upland cotton and can greatly improve the efficiency of salt-tolerance evaluation in massive germplasms in the future.

### Association mapping provides an effective method for identifying favorable alleles

For complex traits, mapping QTLs related to target traits will enable the quick and efficient pyramiding of QTLs by MAS. In cotton, numerous QTLs related to yield, fiber quality, and *Verticillium* wilt resistance have been identified by family-based QTL mapping or association mapping (AM) methods [31]. AM is based on linkage disequilibrium (LD) and uses a sample of lines from the broader breeding population, unrelated by any specific crossing design [8]. A small number of papers reported QTLs related to salt tolerance using AM methods. Recently, Jia et al. [2] reported that 106 microsatellite markers were used to evaluate 323 *G*. *hirsutum* germplasms grown in drought and salt conditions: 15 markers were found to be associated with drought tolerance, and three markers were associated with salt tolerance.

In the present study, 304 upland cotton cultivars were used to carry out AM of ten salt-tolerance related traits using 145 SSRs. At the p<0.05 level, we detected 95 marker-trait associations between 60 SSRs and ten salt-tolerance related traits (Table 2), with 20 marker-trait associations detected simultaneously in two or more traits. The phenotypic effects of each allele were measured (Table 2). Most of the traits simultaneously associated with the common markers belonged to the same factor type, as determined by factor analysis. Seven markers were simultaneously associated with RGR and RGP in the germinative index factor. Five markers were simultaneously associated with RPH and RRDM, and five markers were simultaneously associated with RPH and RSDM in the seedling stage physiological index factor. In the four seedling stage biochemical index factors, two markers were simultaneously associated with three traits.

Among these 60 SSRs, 58 associated with salt-tolerance traits were novel reported loci. Only NAU483 and NAU1042 have been reported previously. Saeed et al. [3] reported that 98 SSR markers were used in the AM of 109 cotton cultivars for ten salt-tolerance traits, and sixteen markers were found to be associated with salt tolerance after salt treatments of different concentrations (100mM NaCl and 200mM NaCl). NAU483 was found to be associated with plant height following 200mM NaCl treatment. In the present study, NAU483 was found to be associated with relative germination percentage. NAU1042 was associated with relative root dry matter. In a previous study that used the (Lumian 97–8 × Sumian 12) F_2_ segregation population, a QTL for the plant height stress coefficient and index at the seedling stage (*qSSPHSC-C6-1*) was identified at marker interval NAU1042-NAU1221, which explained the 12.26% phenotypic variation (PV) [49].

### Perspectives of molecular breeding by design in cotton

Molecular breeding by design was first proposed by Peleman and van der Voort in 2003 [9], which allows the simulation and optimization of the breeding procedure before field experiments and increases predictability in conventional breeding [10]. Several successful examples have been practiced in rice, wheat and cotton crops [50–52]. For improving rice seed quality traits, using 65 chromosome single segment lines (CSSLs) and 82 donor parent chromosome segments with *Oryza japonica* rice variety Asominori as the recipient parent and the *indica* rice variety IR24 as the donor parent, QTLs for two traits, area of chalky endosperm and amylose content, was identified. Further, four target genotypes were designed for low chalky endosperm and high amylose content breeding objectives based on the identified QTLs [50]. In wheat, six major genes involved in plant height, disease resistance, and grain quality traits, and 17 QTLs related to coleoptile length were simultaneously selected by computer simulation for multi-traits improvement [51]. In cotton, pyramiding two QTLs that control high fiber strength by MAS greatly improved the selection efficiency for cotton fiber strength via modified backcrossing breeding methods [52].

With the threat of soil salinization, as well as the contradictions in the land composition required for cotton and grain production in China, the demand of breeding new cultivars with salt-tolerance has increased dramatically. In response to these needs for cotton production, a batch of excellent germplasm resources and molecular markers that can be applied to cotton salt-tolerance breeding, and used to obtain optimal donor combinations for further salt tolerance breeding were obtained in this study. Some of the salt-tolerance molecular markers detected in this study also have an impact on improving fiber quality or pest tolerance, as reported previously; leading to the comprehensive improvement of cotton cultivars. Recently, the whole-genome scaffold sequence of the allotetraploid cotton *Gossypium hirsutum* L. acc. TM-1 was released [53–54]. This laid the foundation for single nucleotide polymorphism (SNP) development through high density, GWASs and can be applied to the improvement of important agronomic traits in upland cotton cultivars through molecular breeding by design.

It is worth noting that there are various types of interactions between multi-QTLs with different origins, such as additive effects, dominance effects, epistasis effects and QTL×environment interactions [55]. Of the 304 accessions used in this study, 176 contained elite alleles more than average, however, only 157 salt-tolerant accessions were identified (43 advanced salt-tolerance cultivars and 114 medium salt-tolerance cultivars). Furthermore, there were also elite salt-tolerant QTLs/genes detected in the salt-sensitive cultivars. Based on this, molecular breeding by design should focus not on only elite allele numbers, but also on interactions between different alleles. In this study, we temporarily assumed that the more pyramiding the favorable alleles were, the better the salt tolerance of accessions for computer simulation, further, we selected 13 cultivars as receptor parents to conduct molecular breeding by design. The optimal donors and cross combinations for pyramiding the largest number elite alleles identified by simulation analysis still require confirmation through field experiments.

In conclusion, in the present study a large quantity of molecular information and a large number of elite germplasms for the improvement of breeding salt-tolerant traits were identified via molecular marker-assisted selection by factor analysis, association analysis, and molecular breeding by design. Further, the optimal crosses for future salt tolerance breeding in cotton were proposed.

## Supporting Information

S1 Fig The distribution of LD among 145 SSR loci on 26 chromosomes in 304 cotton cultivars. (JPG)

S1 Table Cotton cultivars/accessions used in the present study. (XLS)

S2 Table Correlation analysis among ten traits related to salt tolerance. (XLS)

S3 Table Diversity information of 145 SSR loci. (XLS)

S4 Table Elite QTLs for salt-tolerance related traits and the representative cotton cultivars. (XLS)

S5 Table Example for the optimal combination of 13 elite receptors and the selected donors with pyramiding most elite alleles from different cross patterns. (XLSX)
