# Supplementary figures and images for: Evaluation and Exploration of Favorable QTL Alleles for Salt Stress Related Traits in Cotton Cultivars (G. hirsutum L.)
Source: PLoS One. 2016 Mar 4;11(3):e0151076. doi: 10.1371/journal.pone.0151076 (PMC4778925; doi:10.1371/journal.pone.0151076)

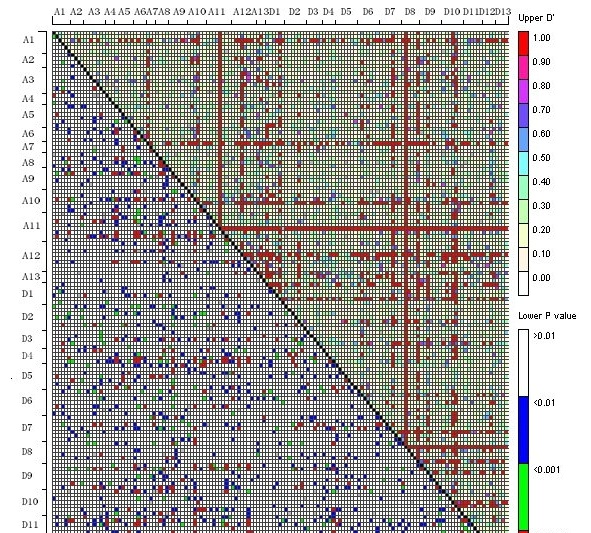

Supplement: S1 Fig — (JPG) [file pone.0151076.s001.jpg]
